# Supplementary material for: Effect of Hydroxyurea on Morphology, Proliferation, and Protein Expression on Taenia crassiceps WFU Strain
Source: Int J Mol Sci. 2024 May 31;25(11):6061. doi: 10.3390/ijms25116061 (PMC11172544; doi:10.3390/ijms25116061)
Supplement: Supplementary file 1 [file ijms-25-06061-s001.zip › Supplem. table S1 unique proteins.pdf]

6 days of HU treatment		
Only in control		
Accession number	Protein	Function
EmuJ_000812800	E3 ubiquitin ligase	Promotes protein ubiquitination and degradation (Zheng et al., 2017
EmuJ_000486000	Ankyrin repeat domain containing protein 6 (ANK6)	Intervene the attachment of transmembrane proteins to cortical cytoskeleton networks (Kim & Oh, 2016)
HU 25 mM		
EmuJ_000855000	Leucyl aminopeptidase (LAP)	Catalyze the removal of N-terminal amino acid residues (Matsui et al., 2006)
EmuJ_000225800	Papilin	embryonic development and interaction with matrix extracellular components (Fessler et al., 2004).
EmuJ_001150700	Nuclear factor 1 A type (NF1)	RNA polymerase III transcription initiation and gene expression (Wang et al., 2000)
EmuJ_000378850	Hypothetical transcript	N/A
EmuJ_000925900	Transformer 2 protein beta TRAB2B	Control pre-mRNA splicing (Xue et al., 2023)
HU 40 mM		
EmuJ_000236500	Major egg antigen	Its part to family of small heat shock proteins (HSP20) (Zhang, Chinese Veterin Sci 50:982–988)
EmuJ_000897200	Nucleolar protein 5/ nol-58	Predicted to be involved in ribosome biogenesis (Kishore et al., 2020)
EmuJ_000723900	3-phosphoinositide-dependent protein kinase 1 (PDK1)	Cell growth and proliferation (Levina et al., 2022)
EmuJ_000215400	N-alpha acetyltransferase 50 NatE catalytic (NAA50)	H4 histone acetyltransferase activity (Fang et al., 2022)
EmuJ_000827900	Malignant t cell amplified sequence 1 (MCTS 1)	Oncogene involved in cell cycle progress (Huang et al., 2021).
EmuJ_001185600	Cullin 1	Molecular scaffold with participation in post-translational modification of cellular proteins involving ubiquitin (Sarikas et al., 2011)
EmuJ_000751300	Uridine-Cytidine Kinase 2 (UCK2)	Responsible for the phosphorylation of uridine and cytidine to their corresponding monophosphate in pyrimidine nucleotides biosynthesis. (Malami & Abdul, 2019).
EmuJ_000736200	Synembrin	A guanine nucleotide exchange factor (Tall et al., 2003)
EmuJ_001128500	CDP-diacylglycerol synthase.	Phospholipid biosynthesis pathways (Blunsom et al., 2020)
3 days of recovery		
Control		
EmuJ_000556100	60S ribosomal protein L23a (RPL23)	Component of the large ribosomal subunit (Warner et al., 2009).
EmuJ_000424500	28S ribosomal protein S7 mitochondrial (MRPS7)	Protein synthesis within the mitochondrion (Koc et al., 1999, Menezes et al., 2015)
EmuJ_000981400	Cullin 2	Scaffold protein (Sarikas et al., 2011)

EmuJ_001123900	Eukaryotic translation initiation factor 3 subunit L (eIF3L)	Initiation and termination of translation, and in ribosomal recycling (Gomes-Duarte et al., 2018)
EmuJ_000344100	Isocitrate dehydrogenase [NAD] mitochondrial (IDH)	Catalyse the oxidative decarboxylation of isocitrate involved in Krebs cycle and cellular homoeostasis (Han et al., 202)
EmuJ_001079800	Multiple inositol polyphosphate phosphatase (MINPP1)	Responsible for the metabolism of inositol hexakisphosphate (InsP6) and inositol 1,3,4,5,6 pentakisphosphate (Yu et al., 2023)
EmuJ_001151300	Protein phosphatase 2C (PP2C)	downregulation of the stress-activated MAP kinase cascades in eukaryotes (Widmann et al., 1999).
EmuJ_000817600	N alpha acetyltransferase 50 NatE catalytic (NAA50)	Enables H4 histone acetyltransferase activity activity (Fang et al., 2022)
EmuJ_000107000	Transmembrane emp24 domain containing protein (TMED/p24)	Regulators of protein transport (Jerome-Majewska et al., 2010)
EmuJ_001131000	Protein phosphatase 1 regulatory subunit 12B PPP1R12B	Enzyme inhibitor activity and phosphatase regulator activity that predicted to be involved in signal transduction (Alliance of Genome)
EmuJ_000440900	AP 2 complex subunit mu 1 AP2M1A	Participles in the link between clathrin to receptors in coated vesicles finally. (Gong et al. 2021)
HU 25 mM		
EmuJ_000850900	Oxysterol-binding protein OSBP	Transports and regulates the metabolism of sterols and phospholipids (Pietrangelo & Ridgway, 2018)
EmuJ_000781600	Venom allergen val protein (VAL)	Involved in host-parasite interactions (Ro Lee et al.,2018)
HU 40 mM		
EmuJ_000056300	Adenylosuccinate lyase (ADSL)	Participles in the purine nucleotide cycle (Crifò et al., 2005)
EmuJ_000744400	DNA2 nuclease/helicase	Involved in multiple DNA metabolic pathways (Zheng et al., 2020)
EmuJ_000440000	T-cell immunomodulatory protein/ Inkn-1	Modulator of T-cell function and involved in cell adhesion. (Kishore R
6 recovery days		
Control		
EmuJ_001000650	Calpain	Intracellular Ca2+-dependent cysteine protease (Sorimachi et al., 2013)
EmuJ_000799700	Ufm1 specific protease 1 (UFSP1)	Regulator of protein homeostasis (Gerakis et al., 2019)
EmuJ_000195300	Glutaredoxin 3 (GLRX3)	Antioxidant enzyme (He et al., 2016)
EmuJ_000879100	Protein phosphatase methylesterase 1 (PPME1)	Improves the ERK and Akt signaling pathways (Kim et al., 2018)
EmuJ_000305200	Signal transducing adaptor molecule STAP-2	Involved in regulation of several intracellular signaling events in immune cells (Matsuda & Oritani, 2021).
EmuJ_000662000	Thioredoxin-like protein Sh3 domain binding glutamic acid rich (SH3BGR)	Expressed in diverse organogenesis in embryonic development in zebrafish (Tong et al.,2016)
EmuJ_000746800	Syntaxin 1a	Mediates targeted vesicular fusion at the synapse (Ghezzi et al., 2011)

EmuJ_001019100	U1 small 70 kDa (snRNP70)	nuclear ribonucleoprotein (Fan et al., 2021)
HU 25 mM		
EmuJ_000660400	N-cadherin (CDH2)	Role in neural tissue (László & Lele, 2022)
EmuJ_000847400	ATP dependent zinc metalloprotease (FtsH)	protein quality control regulation (Sauer et al., 2004, Bieniossek et al., 2006)
EmuJ_000437000	Ribonucleoside diphosphate reductase subunit (RRM1)	Catalyze the de novo conversion of nucleotides to deoxynucleotides in all organisms (Greene ET AL., 2020)
EmuJ_000700600	Glutathione synthetase (GS)	Enzyme in the glutathione biosynthesis pathway (Lu, 20212)
EmuJ_000158800	Aconitate hydratase	Enzyme that catalyses the stereo-specific isomerization of citrate to isocitrate (Beinert & Kennedy, 1993).
EmuJ_000245100	Endoplasmic reticulum oxidoreductin 1 (Ero1)	Protein folding in endoplasmic reticulum (Araki et al., 2012)
40 mM		
EmuJ_000057700	Prominin	localizes to membrane protrusions (Shmelkov et al., 2005)
EmuJ_000869500	Oxysterol binding protein 1 OSBP1	Transports and regulates the metabolism of sterols and phospholipids (Pietrangelo & Ridgway, 2018)
EmuJ_001016100	Secretory carrier-associated membrane protein SCAMPs	Implicated in membrane trafficking (Hubbard et al., 2000).
EmuJ_000443000	Nucleoporin seh1 A	Component of the nuclear pore complex, necessary for normal nuclear morphology (Dokudovskaya et al., 2011, Rout et al., 2000).
EmuJ_000694380	40S ribosomal protein S23 RPS23	Part of the machinery charged to protein synthesis in cells (Ma et al., 2020)
EmuJ_000523600	Splicing factor 3b subunit 2 SF3B2	Required for accurately recognizing the branch point of pre-messenger RNAs during splicing process (Goals et al.,2003,
EmuJ_001115100	Ankyrin repeat containing AK6	Intervene the attachment of transmembrane proteins to cortical cytoskeleton networks (Kim & Oh, 2016)
EmuJ_001105300	Zw10 protein	Component of the mitotic spindle checkpoint (Li et al., 2020)
EmuJ_000766600	Peptidase inhibitor 16 PI16	Involved in negative regulation of peptidase activity (alliancegenome.org)
