# Supplementary material for: Effect of Hydroxyurea on Morphology, Proliferation, and Protein Expression on Taenia crassiceps WFU Strain
Source: Int J Mol Sci. 2024 May 31;25(11):6061. doi: 10.3390/ijms25116061 (PMC11172544; doi:10.3390/ijms25116061)
Supplement: Supplementary file 1 [file ijms-25-06061-s001.zip › Supplementary figure S1.pdf]

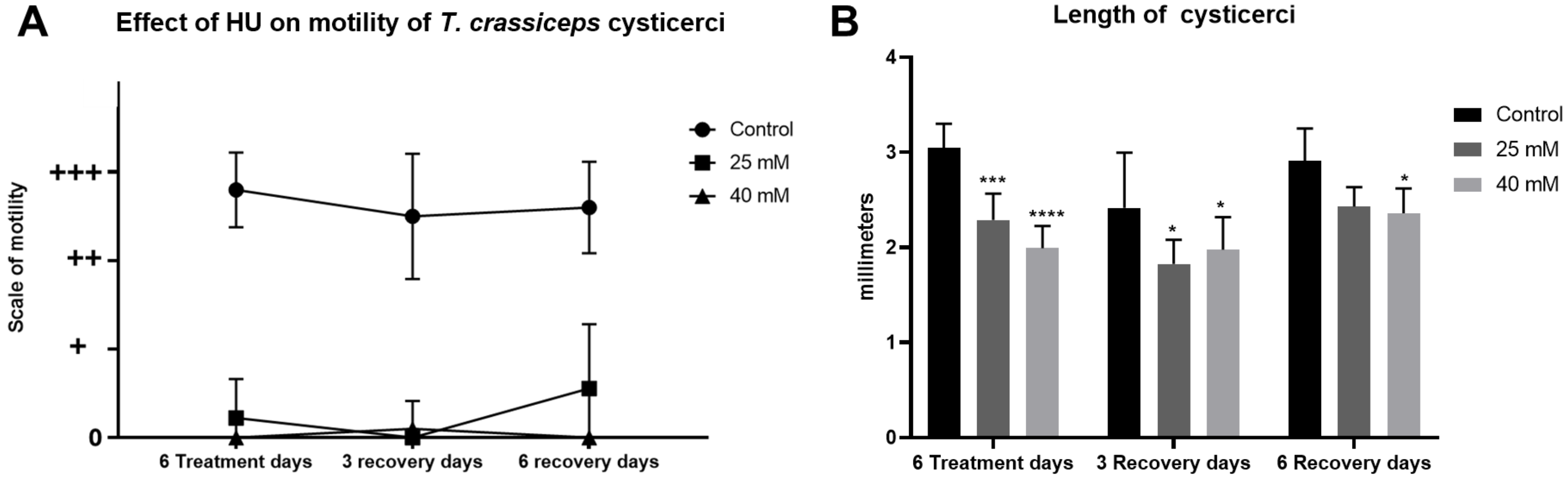

Supplementary figure 1. Effect of HU on the motility and size of *T. crassiceps* cysticerci. **A)** The mobility was evaluated based on the videos acquired in the stereomicroscope. A statistics analysis was made in Prisma software with a normality analysis Shapiro-Wilk test with α=0.05, was realized a two-way ANOVA followed by Dunnet tests: all the treatments have a Dunnett's multiple comparisons test of p = <0.0001 in comparison with the controls without treatment. **B)** The length of each cysticerci was measured in Fiji software based on microscopy scale. A statistics analysis was made in a Prisma software with a normality analysis Kolmogorov-Smirnov test with α=0.05, was realized a two-way ANOVA followed of Dunnet tests: **** p = <0.0001, *** p= 0.001, * p= 0.01.
