# Supplementary material for: Effect of Hydroxyurea on Morphology, Proliferation, and Protein Expression on Taenia crassiceps WFU Strain
Source: Int J Mol Sci. 2024 May 31;25(11):6061. doi: 10.3390/ijms25116061 (PMC11172544; doi:10.3390/ijms25116061)
Supplement: Supplementary file 1 [file ijms-25-06061-s001.zip › supplementary figure S2.pdf]

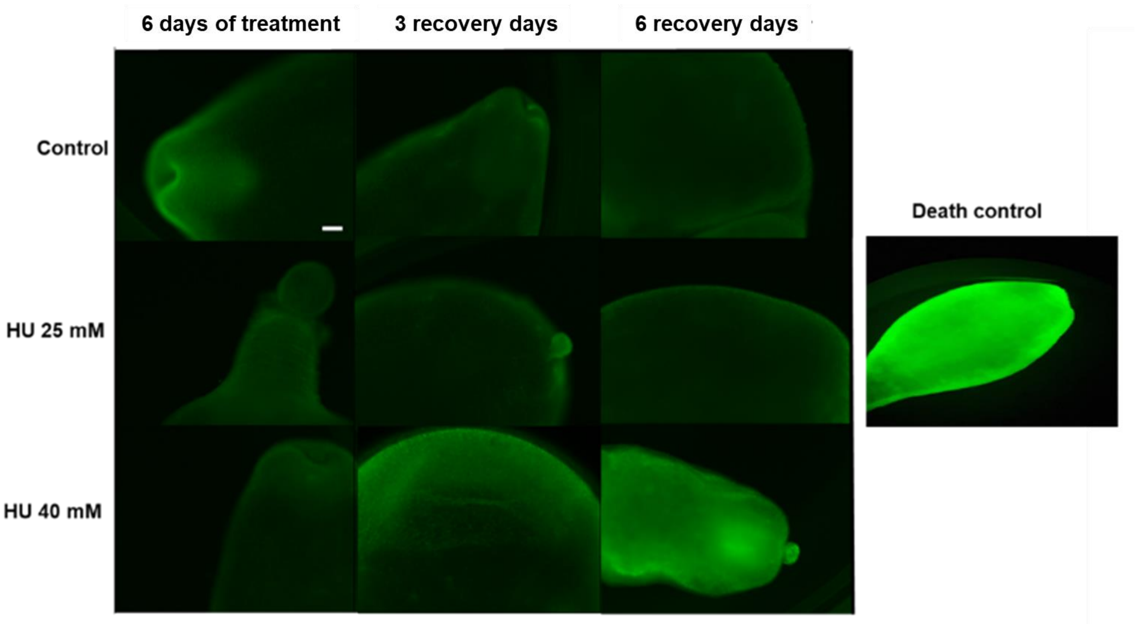

Supplementary Figure 2. Viability assay with Sytox Green marker. The fluorescence marker increased in cysticerci treated with the 40 mM HU independently of the days of recovery. Scale bar represented 100 µm.
